# Proteogenomic links to human metabolic diseases

**DOI:** 10.1038/s42255-023-00753-7

**Published:** 2023-02-23

**Authors:** Mine Koprulu, Julia Carrasco-Zanini, Eleanor Wheeler, Sam Lockhart, Nicola D. Kerrison, Nicholas J. Wareham, Maik Pietzner, Claudia Langenberg

**Affiliations:** 1 MRC Epidemiology Unit, University of Cambridge School of Clinical Medicine, Institute of Metabolic Science, Cambridge, CB2 0QQ, UK; 2 MRC Metabolic Diseases Unit, Wellcome-MRC Institute of Metabolic Science, University of Cambridge, Cambridge, UK; 3 Computational Medicine, Berlin Institute of Health at Charité-Universrtˆtsmedizin Berlin, 10117 Berlin, Germany; 4 Precision Healthcare University Research Institute, Queen Mary University of London, UK

## Abstract

Studying the plasma proteome as the intermediate layer between the genome and the phenome has the potential to identify new disease processes. Here, we conducted a *cis*-focused proteogenomic analysis of 2,923 plasma proteins measured in 1,180 individuals using antibody-based assays. We 1) identify 256 unreported protein quantitative trait loci (pQTL), 2) demonstrate shared genetic regulation of 224 cis-pQTLs with 575 specific health outcomes, revealing examples for important metabolic diseases, like gastrin releasing peptide as a potential therapeutic target for type 2 diabetes, 3) improve causal gene assignment at 40% (n=192) of overlapping risk loci, and 4) observe convergence of phenotypic consequences of cis- pQTLs and rare loss-of-function gene burden for twelve proteins, like *TIMD4* for lipoprotein metabolism. Our findings demonstrate the value of integrating complementary proteomic technologies with genomics even at moderate scale to identify novel mediators of metabolic diseases with the potential for therapeutic interventions.

## Introduction

Rare and common sequence variation across the genome contributes to the risk of most human diseases investigated to date (1). However, the translation of the many established and emerging genome-to-phenome links is limited by the uncertainty around the underlying causal genes. This presents a major limitation for experimental follow-up, mechanistic understanding, and use of the emerging genomic evidence in drug development. Different approaches, such as integration of tissue-specific gene expression data (2), experimentally derived functional genomic data such as ChIP-seq or ATAC-seq (3), or functional characterization of candidate variants using CRISPR screens in cellular models (4) have been used to address this gap and to identify likely causal genes at risk loci. However, complex regulatory processes take place at each stage of transcription and translation, which leads to low to moderate correlation between transcript and protein abundance, and cellular models can only approximate complex human biology. Compared to these methods, a proteogenomic approach has the advantage of focusing on the biologically active entity - the protein.

The development of broad-capture proteomic assays, targeting thousands of proteins in parallel, now enables proteogenomic approaches which can efficiently identify causal genes by systematically testing for a shared genetic regulation of protein levels or function and disease susceptibility. This has catalyzed substantial advances in the identification of a) causal genes and proteins underlying established disease ‘loci’, and b) molecular ‘hubs’ that connect the genome not to one but many diseases through the encoded protein (5–18). Previous large-scale proteogenomic studies covering thousands of proteins have almost exclusively used aptamer-based assays (10, 11, 15, 16). Correlations of protein measures from aptamer versus antibody-based technologies have been shown to vary widely, and proteogenomic results are concordant for around only 65% based on around 900 overlapping proteins targets (14). To date, antibody-based proteomic assays have only been available for selected protein panels at scale (6, 7, 9, 13, 14, 18), but this is changing with the availability of the Olink® Explore 1536 and Olink® Explore Expansion assays measuring ˜1,400 proteins each.

The UK Biobank Pharma Proteomics Project (UKB-PPP) project which measured ˜1,400 proteins using Olink® Explore 1536 assay in over 50,000 participants successfully demonstrated the power of scaling up by cataloguing over 10,000 mainly novel protein quantitative trait loci (pQTLs), including high-impact rare variants (19). However, these studies provided few insights about the translational potential of pQTLs to systematically inform candidate gene annotation at known risk loci and more importantly, to reveal novel biological roles of proteins for human health at scale. UKB-PPP and others did demonstrate that genuine and biologically relevant protein quantitative trait loci (pQTL) can be discovered in as few as hundreds of individuals (14, 17, 20), suggesting that broader proteomic coverage in even small-scale proteogenomic studies can make substantial advances to the understanding of diseases if integrated with genome-wide association statistics from bespoke, large-scale studies on diverse diseases and broader health characteristics.

Here, we generate antibody-based proteomic data using the Olink® Explore 1536 and Explore Expansion assays to capture 2,923 proteins in 1,180 individuals. We perform genetic fine-mapping at protein coding genes (±500kb) and enhance the understanding of disease mechanisms by systematically integrating cis-pQTLs with thousands of diseases and health measures to (a) refine the candidate causal gene assignment at existing disease susceptibility loci at scale and (b) identify novel disease mechanisms in phenome-wide colocalization analyses.

## Results

### Fine-mapping cis-pQTLs for 2,923 protein targets

**Figure 1:**
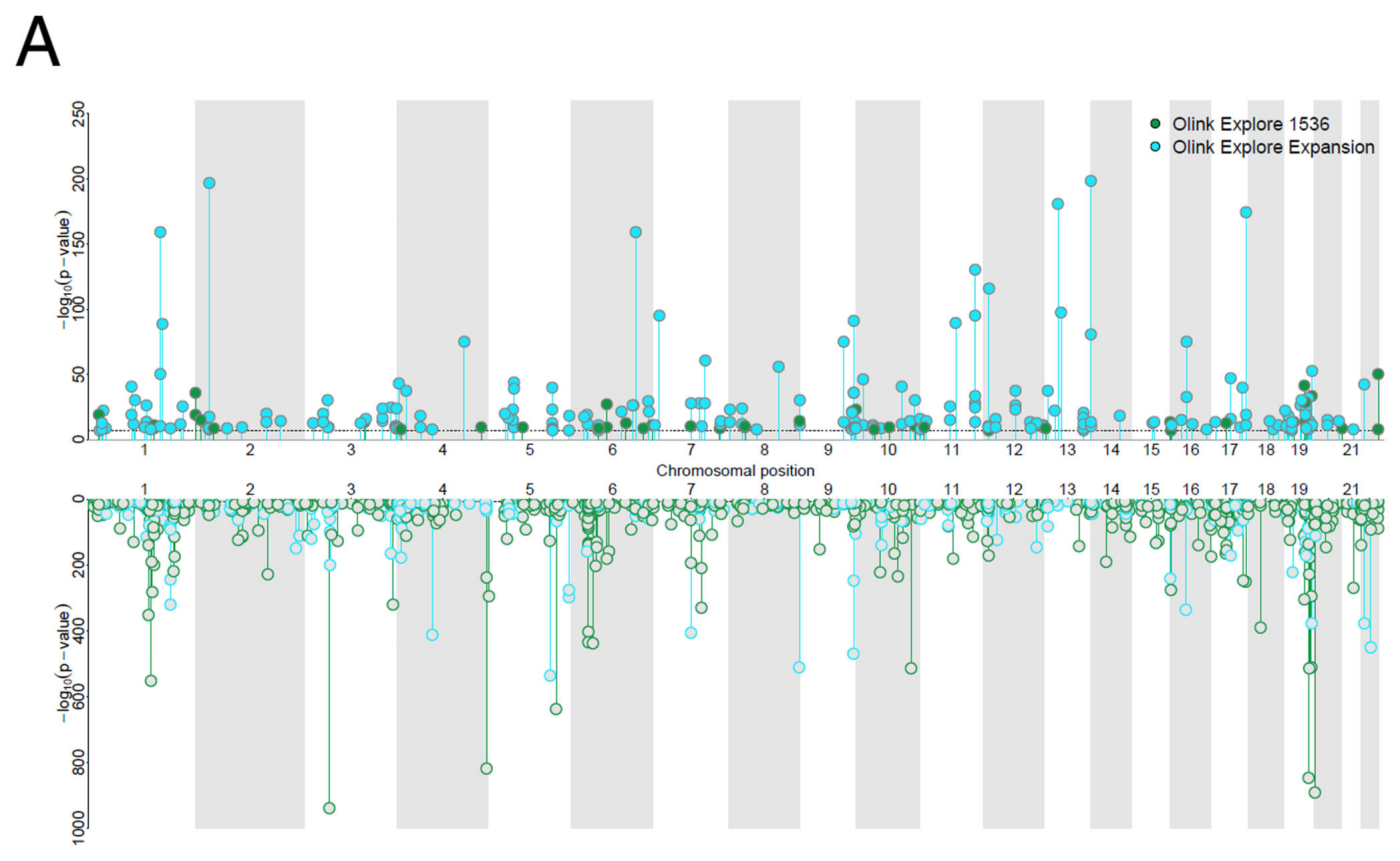

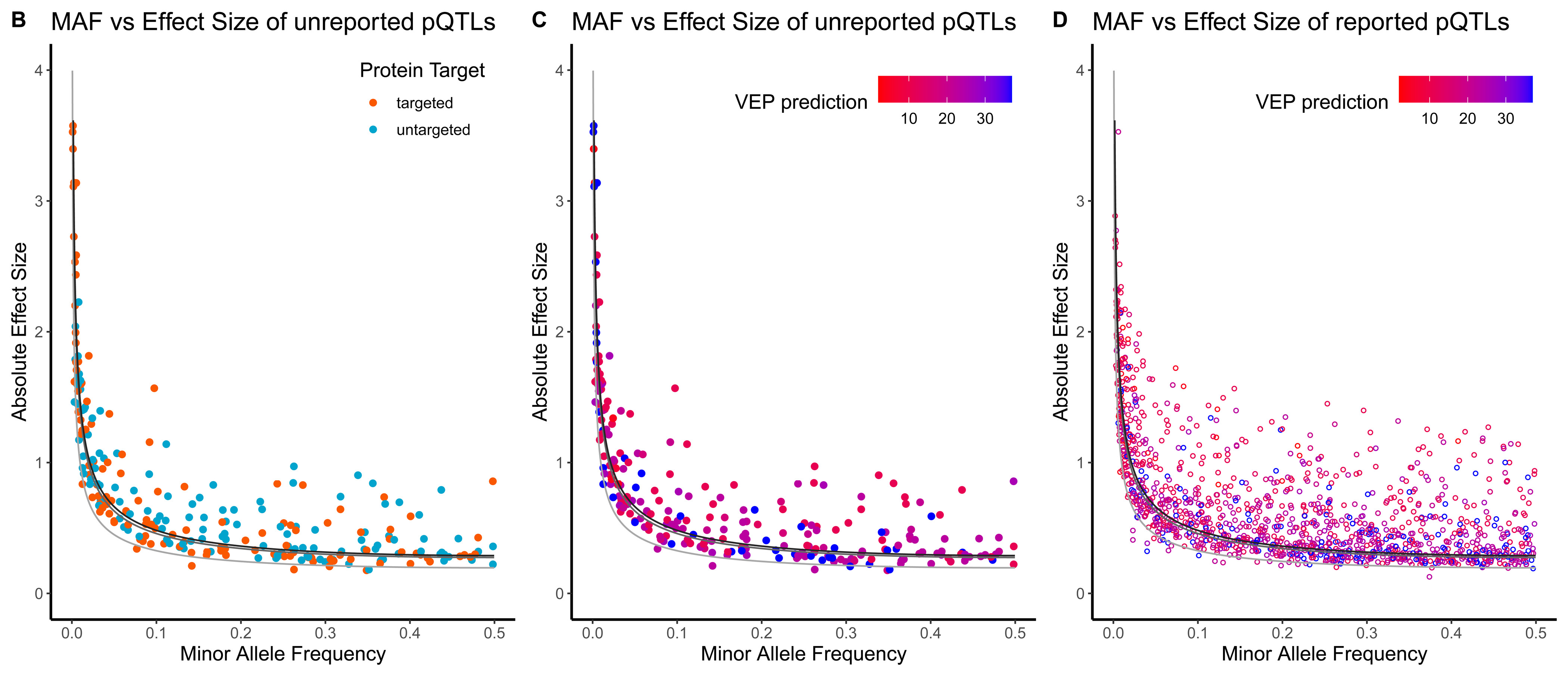
Genetic associations of 2,923 proteins measured by the Olink Explore 1536 and Olink Explore Expansion platforms in 1,180 individuals. Previously unreported and reported pQTLs are represented with a filled and hollow circle, respectively. Only the variants which are genome-wide significant (p-value<5x10^-8^) in the joint model are presented. **A. Miami plot representing the independent lead cis-pQTLs identified through Bayesian fine-mapping for 914 unique proteins.** Shown are p-values from a linear regression model modelling all identified credible set variants for a given protein target jointly. *Top:* Lead cis-pQTL signals unreported to date. *Bottom:* Lead cis-pQTL signals which were in linkage disequilibrium (LD; r^2^>0.5) with a previously reported pQTL. **B. Minor allele frequency vs effect size of unreported pQTL signals, coloured by whether the protein has previously been targeted.** Unreported pQTL signals for a previously targeted protein are coloured grey and those for a previously untargeted protein are coloured orange. **C. Minor allele frequency vs effect size of unreported pQTL signals, coloured by most severe variant consequence prediction.** The colour coding represents the most severe Variant Effect Predictor (73) consequence of the lead cis-pQTL, or variants in LD (r^2^>0.6) within the protein encoding gene. The most severe consequence is coloured red (Ensembl consequence rank = 1) and the least severe consequence is coloured blue (Ensembl consequence rank = 37). **D. Minor allele frequency vs effect size of reported pQTL signals, coloured by most severe variant prediction.** The colour coding represents the most severe Variant Effect Predictor consequence of the lead cis-pQTL, or variants in LD (r^2^>0.6) with the lead cis-pQTL within the protein encoding gene. The most severe consequence is coloured red (Ensembl consequence rank = 1) and the least severe consequence is coloured blue (Ensembl consequence rank = 37). Lines are power curves which represent 25% (light grey), 90% (medium grey) and 95% (dark grey) power from the bottom to the top, respectively in our study with 1,180 participants for inverse rank normalized protein level measurements.

We adopted a Bayesian fine-mapping strategy to identify proximal acting genetic variants (cis-pQTLs, ±500kb around the protein coding gene) that were associated with plasma abundance of 2,923 proteins measured in 1,180 participants of the EPIC-Norfolk cohort (21) (Supplementary Table 1–2). We identified a total of 1,553 independent credible sets for 914 unique protein targets for which sentinel variants reached genome-wide significance (p<5x10^-8^) when modelled jointly at each protein coding locus (Fig. 1A, Supplementary Table 3). The number of independent credible sets for each protein target ranged between one and eight (mean=1.64, IQR=1-2), illustrating wide-spread allelic heterogeneity at protein coding loci. This included 256 unreported credible sets (Fig. 1B), 236 of which successfully replicated in an independent test set (Supplementary Table 3) (5–18). We further observed a high-replication rate for 590 proteins overlapping with the UKB-PPP effort (89.9%, 910 out of 1,013 Olink® Explore 1536 cis-pQTL credible sets). Notably 125 of the novel signals were for 101 previously targeted proteins, the majority of which (n=92 proteins) have been targeted using non-antibody-based technologies in samples sizes up to 30 times larger than ours (10, 11, 15, 16) (Fig. 1B).

We observed that the distributions of effect sizes and minor allele frequencies for unreported cis-pQTLs were comparable to the 1,297 (83.5%) successfully replicated cis-pQTLs (5–18) (Supplementary Table 3), illustrating that complementary proteomic technologies can still identify genetic variants that would have been anticipated to be seen in previous studies with much larger sample sizes (Fig. 1A-D). This included 34 previously unreported cis-pQTLs with a minor allele frequency (MAF) above 5% with large absolute effect sizes (range 0.5-1.57 s.d. per allele), half of which (n=19) are unlikely to be a result of altered epitope binding of the affinity reagent and hence illustrate a strong genetic control for selected protein targets. In general, the identified cis-pQTLs explained a median of 10.1% of the variance in adjusted protein abundances (IQR: 4.9% - 22.4%), with lead-cis pQTLs explaining over 50% of the variance for 59 protein targets (Supplementary Table 4).

Proteins with at least one significant cis-pQTL were enriched for characteristics of secreted proteins, like the presence of disulfide-bonds (odds ratio (OR) [95%-CI]: 4.47 [3.78-5.31]; p-value=3.0x10^-74^) or glycosylation sites (OR [95%-CI]: 2.22 [1.79-2.75]; p-value=1.1x10^-13^), but depleted of sites for posttranslational modifications that are important for intracellular signaling, like phosphorylation (OR [95%-CI]: 0.42 [0.34-0.54]; p-value=5.5x10^-14^) or ubiquitination (OR [95%-CI]: 0.30 [0.19-0.45]; p-value=4.2x10^-11^).

For more than half of the protein targets (n=532) with at least one cis-pQTL, we observed strong evidence of colocalization (PP>80%) between a cis-pQTL and the corresponding gene expression QTL (eQTL) signal in at least one out of 49 tissues of the GTEx resource (Supplementary Table 5). These results suggest altered expression of protein coding genes in one or multiple tissues as a major source for cis associations observed with plasma protein levels.

We finally tested whether any of the sex differences in the effect of all the identified cis-pQTLs and detected only four variants that passed the multiple testing correction (p<0.05/1553; Supplementary Table 6). This included sex-differential effects of proteins most highly expressed in the reproductive system like TEX101 (rs35033974, p-valuesex-interaction=1.11x10^-8^, beta_women_ [95% CI] = 0.19 [0.08 – 0.30], beta_men_= 0.67 [0.55 - 1.88]; testis) and PAEP (rs783768, p-value_sex-interaction_=4. 18x10^-10^, beta_women_ [95% CI] = 0.66 [0.48 – 0.69], beta_men_= 1.16 [0.17 – 1.26]; endometrium), as well as two proteins (KLK4 and COL28A1) involved in collagen chain trimerization. While not tested here, previous studies with larger sample sizes did not observe any genetic sex-differential effects on the X chromosome (15).

### From genome to phenome via the proteome

**Figure 2:**
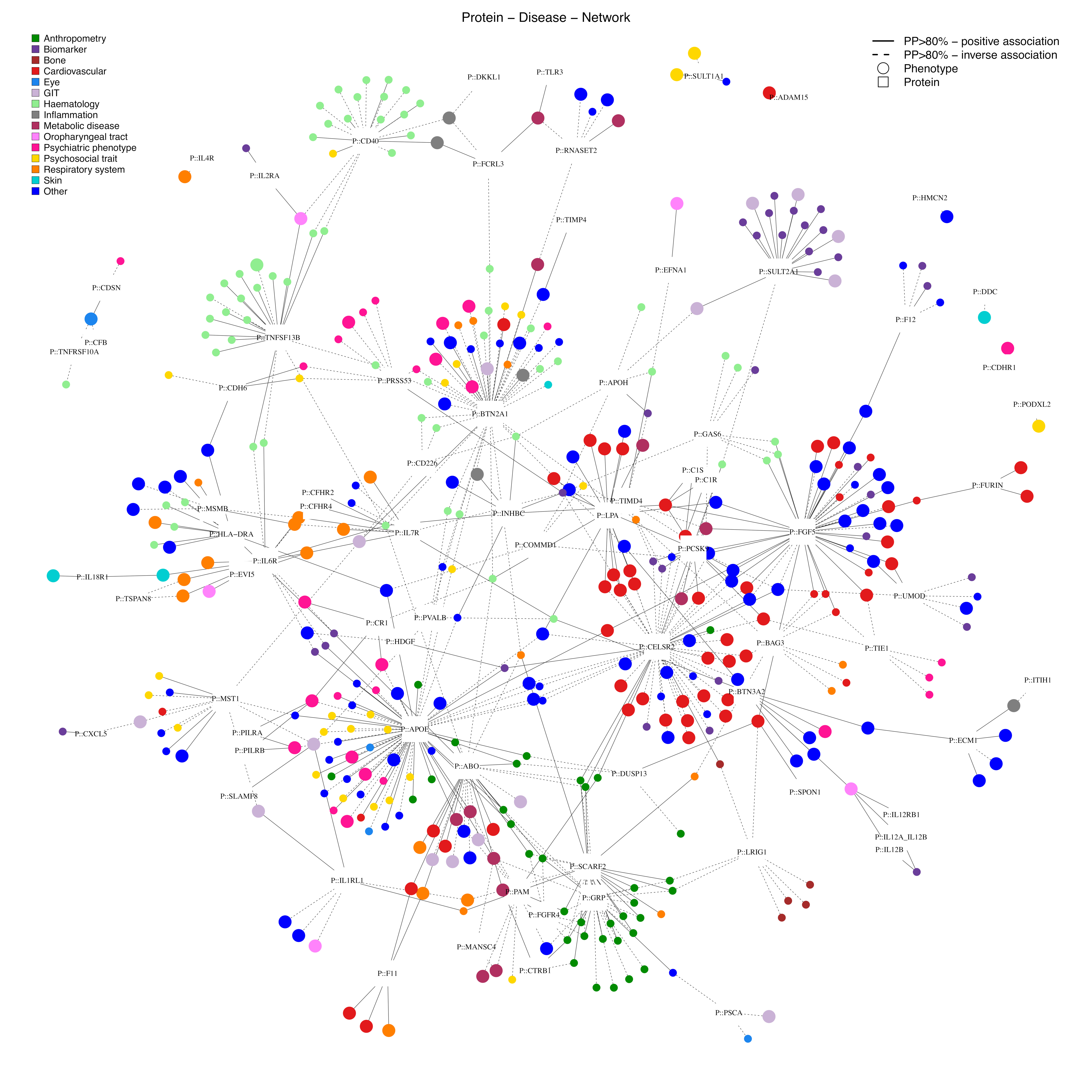
Protein – disease network. Results from phenome-wide colocalization at protein coding loci (±500kb) are shown. For simplicity, only proteins with at least one binary outcome (i.e., mainly diseases) association are included. Proteins are presented with a square, binary outcomes are presented with large circles, and continuous outcomes are presented with small circles. The colour for the circles present the trait category. Edges between proteins and phenotypes represent strong evidence for a shared genetic signal (PP>80% and LD between regional sentinel variants >0.8). Effect directions are indicated by the line type (solid = higher protein abundance, increased risk, dashed = higher protein abundance, reduced risk) and derived based on the lead cis-pQTL at the corresponding locus. The full list of colocalization results can be found in Supplementary Table 7 and results can be viewed in full resolution in Cytoscape session provided in Supplementary Data 1. Abbreviations: GIT, gastrointestinal tract.

The genome is linked to the phenome via the proteome and the translational potential of pQTLs is due to their ability to link insights about the genetic regulation of protein levels and function to diseases (15). We identified 1,110 robust protein – phenotype pairs (Fig. 2; posterior probability [PP] > 80% of a shared genetic signal) comprising 224 protein targets for 575 unique traits by systematically testing for a shared genetic architecture at protein coding loci (±500kb) across the phenome (Supplementary Table 7). This included well-described examples, such as UMOD and kidney disease or established drug targets like PCSK9 and LDL-cholesterol. Notably, we observed evidence for phenotypic consequences of cis-pQTLs for a total of 93 protein targets, which were not observed in our previous study (15), with almost ten times higher sample size but using an aptamer-based technology, clearly highlighting the value of performing proteogenomic studies even for protein targets captured in massive scale studies.

**Figure 3:**
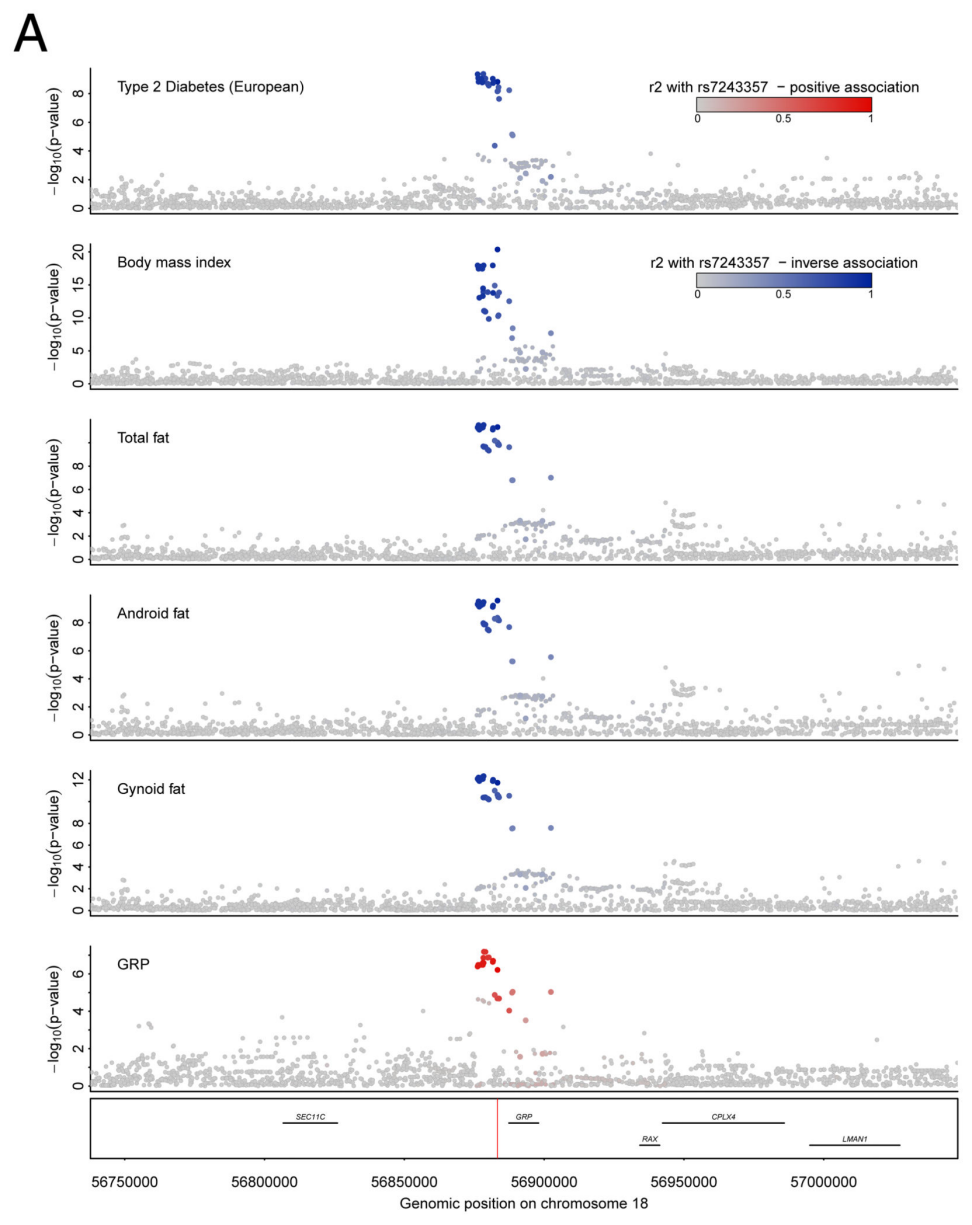

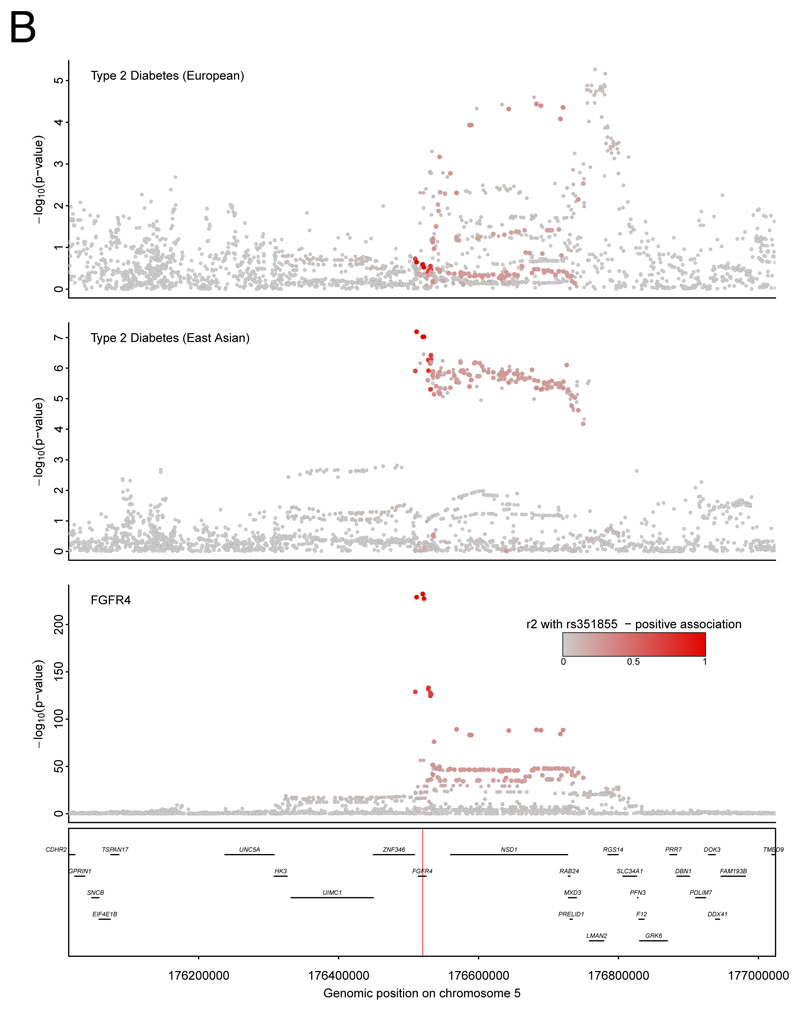
Stacked regional association plots for the multi-trait colocalization. Linear and logistic regression models were used to obtain summary statistics presented in this figure. **A. Stacked regional association plots for the multi-trait colocalization of the GRP cis-pQTL with gynoid fat, android fat, total body fat, body mass index and type 2 diabetes.** The top candidate SNP highlighted by multi-trait colocalization (rs7243357) and lead cis-pQTL for GRP (rs1517035) are in strong LD (r2=0.8). Gynoid fat, android fat and total body fat phenotypes are based on UK Biobank and were analysed in-house using BOLT-LMM (80). **B. Stacked regional association plot the multi-trait colocalization of the FGFR4 cis-pQTL with type 2 diabetes in East Asian populations.** Red colouring represents a positive effect direction in reference to the protein increasing allele for GRP whereas blue represent an inverse association. The hue of the colour represents the strength of r^2^ representing the LD structure, as indicated on the legend. European Type 2 diabetes summary statistics were obtained from dbGAP Million Veteran Program (MVP) European subset (ncases= 148,726, ncontrols= 965,732) (25). East Asian Type 2 diabetes summary statistics were obtained from Mahajan et al (2022) (ncases= 56,268, ncontrols= 227,155) (24). The body mass index summary statistics were obtained from Pulit et al. (2019) (n=806,834) (81).

One of the examples was gastrin releasing peptide (GRP, encoded by *GRP*), for which we observed strong evidence of colocalization (PP=82.5%) between plasma levels and type 2 diabetes (T2D) risk at an established genome-wide association study (GWAS) locus (18q21) for which different genes had been prioritized, including *SEC11C*, *GRP*, and *MC4R* (22–24). The GRP-increasing G-allele of the lead cis-pQTL (rs1517035; MAF=0.18) was associated with a reduced risk for T2D in the largest T2D study (25) (OR [95% CI] = 0.96 [0.95-0.98], p-value=7.8x10^-10^). GRP is a neuropeptide named for its ability to stimulate secretion of the gastric acid secretagogue, gastrin, in the stomach (26, 27), but it is likely involved in other metabolic pathways. We obtained strong evidence that GRP likely mediates T2D risk via an effect on overall obesity based on the convergence of evidence from mice studies (28, 29), (30), human trials (31) and human genetic data from this study. Briefly, we established a shared genetic signal between plasma GRP, body mass index and fat, and T2D risk using multi-trait colocalization with coherent effect directions (Fig. 3). GRP induces satiety in mice via its cognate GRP receptor (*Grpr*) (28, 29) and mice lacking *Grpr* show impaired glucose tolerance after gastric glucose administration (30) and gain excess body weight under *ad libidum* conditions (29). These observations have been corroborated by human trials, in which treatment with human recombinant GRP (hrGRP) led to weight loss through reduced food intake (31). In summary, our results motivate investigations into hrGRP for appetite control and body weight lowering to possibly assist in T2D management and remission, an approach similar to recently implemented treatment strategies targeting incretins, like GLP-1, and associated receptors, with preliminary evidence of an additive effect in rats (32).

Several risk loci for T2D have been reported to be specific to certain ancestries (22–24). In the absence of strong differences in allele frequencies, such ancestry specific effects could be caused by a variety of different factors, including environmental factors such as dietary intake. We obtained robust evidence that *FGFR4* is the candidate causal gene at the East Asian-specific *FGFR4-NSD1* risk locus supported by a high PP of 97% for a shared genetic signal with plasma levels of the gene product fibroblast growth factor receptor 4 (FGFR4) and cross-ancestral conserved LD between regional sentinel variants (r^2^>0.96; Fig. 3). The protein-increasing A-allele of the lead cis-pQTL (rs351855, beta= 1.01, p-value=9.8x10^-234^, EAF_European_=0.30, EAF_EastAsian_=0.46) was associated with an increased risk for T2D (OR [95%-CI] = 1.28 [1.17 – 1.40], p-value=1.1x10^-7^) in East Asians (24). Candidate gene studies have implicated rs351855 (p.G388R) in cancer susceptibility (33–35), and subsequent mechanistic studies showed a gain of function of the mutant FGFR4 by binding transducer and activator of transcription 3 (STAT3) (36). While we found no evidence for an association to cancer, there are different studies that support our observation of FGFR4 in T2D-related pathways including hepatic glucose, bile, and lipid metabolism, and possibly insulin signaling in a diet-dependent manner (37–40). Briefly, *Fgfr4^-/-^* mice fed a normal chow diet exhibit insulin resistance and impaired glucose tolerance compared to wild-type controls, however, this difference is not observed in high-fat diet fed mice where both groups showed signs of insulin resistance. A similar masked genetic effect by a high-fat diet is seen with the mutant protein in mice and small observational studies in humans (41). The ability of diet to obscure genetic effects may explain the ancestral-specific effect in the absence of strong differences in allele frequencies, with high-fat diet conditions being substantially more common in Western-style countries of predominantly European ancestry compared to East Asia (42), in particular Japan, in line with Biobank Japan (p-value_T2D_=7.6x10^-11^) being the largest contributing population to the East Asian T2D meta-analysis (24).

### Proteogenomic annotation of genes at reported risk loci

Annotation of the candidate causal genes at disease susceptibility loci is the major bottleneck in the translation of GWAS into biological and possibly clinical insights (43). We exploited the genomic proximity between cis-pQTLs and the protein coding gene for gene annotation by systematically overlapping identified credible sets in this study with reported risk loci (p<5x10^-8^) from the GWAS catalog (downloaded on 23/03/2022; (1)).

**Figure 4:**
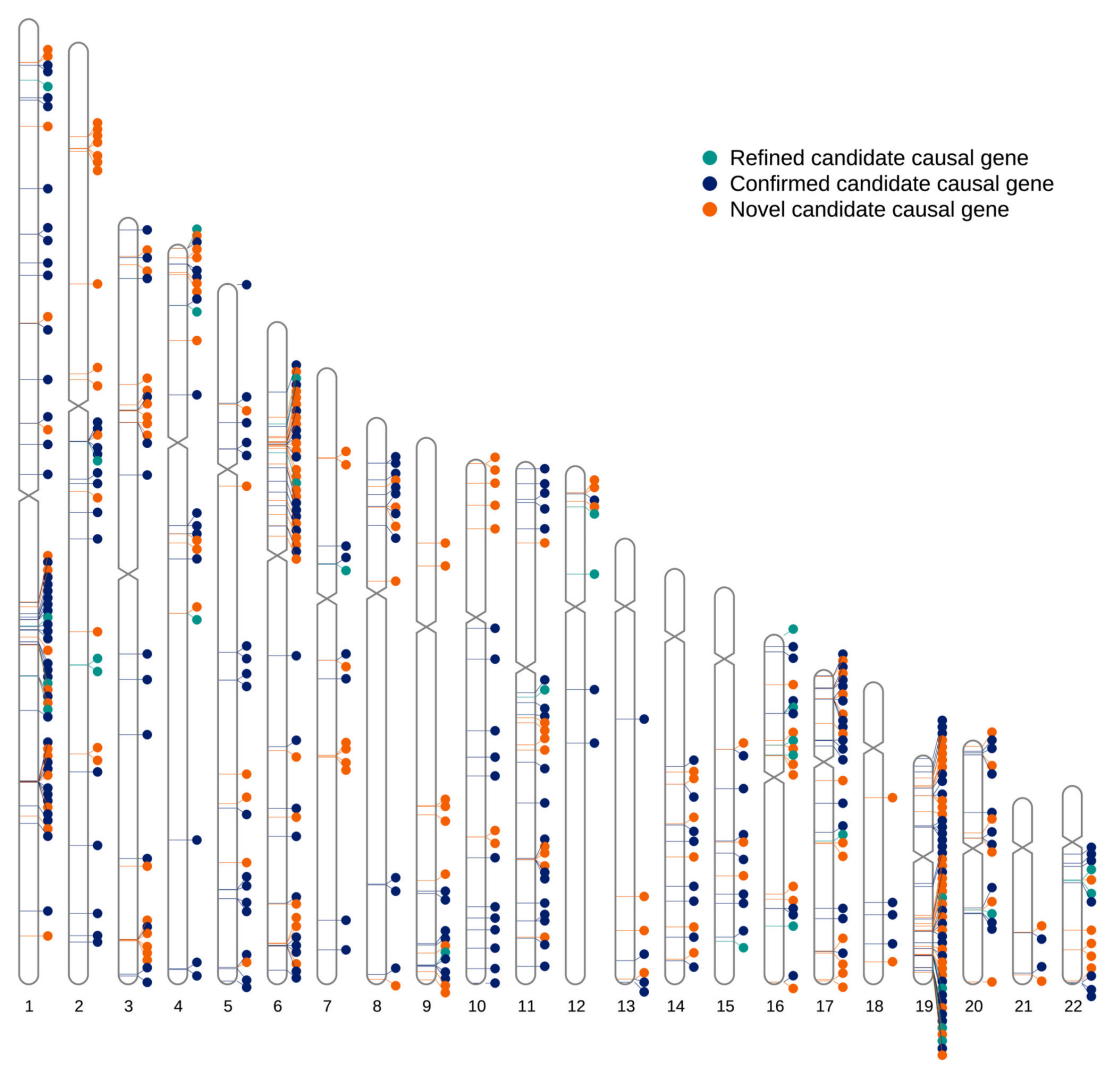
Candidate causal gene assignment at reported GWAS loci using pQTLs. The marked genetic locations on the human karyotypes (chromosomes 1-22) only present the existing GWAS risk loci which overlapped with pQTL loci (n=480). The locus is coloured orange if the pQTL provides a novel candidate causal gene assignment for one or more traits, light blue if it refines a candidate causal gene from a longer list of reported or closest genes, and dark blue if it confirms the candidate causal gene assignment provided by the GWAS.

We identified 480 credible sets targeting 395 unique proteins (43.2% of all, 914 unique protein targets) for which the lead cis-pQTL or a proxy (r^2^>0.8) had been reported as regional lead signal for one or more of 5,391 collated traits in the GWAS catalog (Fig. 4 and Supplementary Tab. 8,). This included 236 unique protein targets (59.7%) that had also matching evidence for colocalization with gene expression events in at least one tissue, providing additional confidence in candidate causal gene assignment, whereas the reaming ones demonstrate the importance of additional functional genomic layers to facilitate causal gene assignment.

For 40% (n=192) of the thereby annotated loci, we prioritized a gene that was different from the one originally reported, of which 50% (n=96) were not the gene nearest to the GWAS sentinel variant. We further refined a longer list of putative causal genes to a single one for an additional 31 cis-regions (6.5%). While systematic testing for a shared genetic architecture using statistical colocalization was not possible due to the general lack of genome-wide summary statistics, about half (49.6%) of the protein targets were also highlighted in our colocalization analysis.

These results exemplify the unique potential of cis-pQTLs for gene annotation of loci reported across diseases and traits related to human health (Fig. 4 and Supplementary Tab. 8). For example, we identify *DKKL1* as a candidate causal gene for multiple sclerosis (MS), potentially through a role in B-cell hyperactivity, which may provide late genetic evidence for depletion of B-cells being one of the most effective treatments for MS, a therapeutic strategy that originally emerged from clinical and neuropathological studies (44, 45) (see Supplementary Note 1).

**Figure 5:**
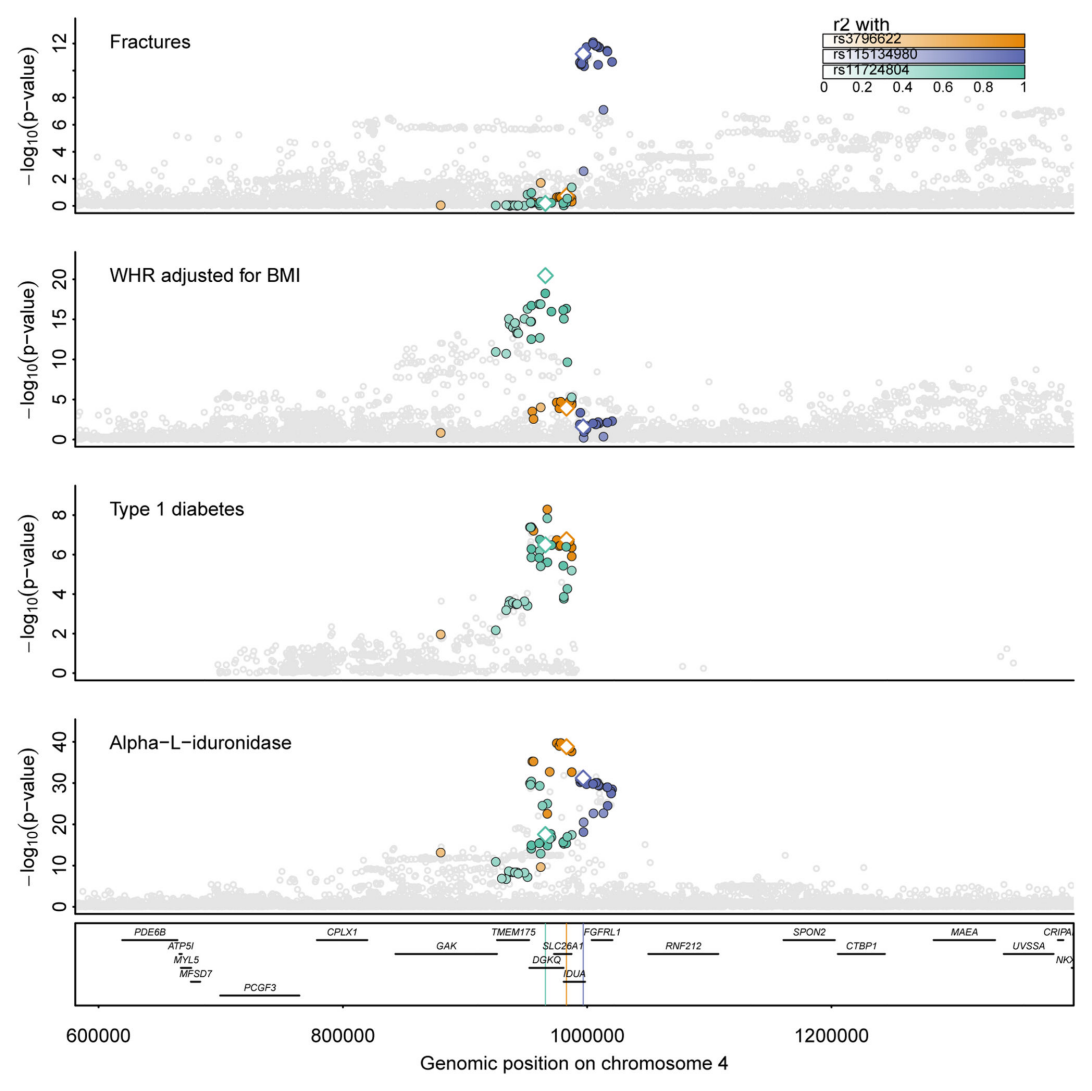
Allelic heterogeneity at protein coding loci translates into distinct phenotypic consequences at *IDUA.* Regional associations plots centered around IDUA (±400kb) for plasma alpha-L-iduronidase levels, type 1 diabetes (50), waist-to-hip ratio (WHR) adjusted for body mass index (BMI) (48), and risk of fractures (46). Shown are association statistics (p-values) from genome-wide association analysis, obtained from linear and logistic regression models. Single genetic variants were coloured based on LD with three distinct cis-pQTLs (rs3796522 – orange; rs115134980 – purple; rs11724804 – green). Lead cis-pQTLs are highlighted by hollow diamonds.

Multiple independent genetic variants being associated with the same protein target at the same locus, so-called allelic heterogeneity, provides the highest confidence in gene assignment but can also highlight differential biological roles for the same protein. We observed 73 such protein targets with two or more credible sets including distinct GWAS variants for related and unrelated traits. For example, we observed a segregation of phenotypes across distinct cis-pQTLs for alpha-L-iduronidase encoded at *IDUA*. Briefly, three out of four detected credible sets contained GWAS risk loci or strong proxies (r^2^>0.8) for fractures for fractures (rs115134980; MAF=16.1%; OR [95% CI] = 0.94 [0.92 − 0.96], p-value=7.4x10^-12^) (46) and bone mineral density (rs115134980; MAF=16.1%; beta=0.07, p-value=1.8x10^-17^) (47), waist-to-hip ratio adjusted for BMI (48) (rs11724804; MAF=44.7%; beta=-0.017, p-value=7.6x10^-21^) and inflammatory diseases (49), as well as type 1 diabetes (50) (rs3796622; MAF=35.2%; OR [95% CI] = 0.93[0.90-0.96], p-value=1.7x10^-7^) (Fig. 5). Alpha-L-iduronidase is essential for the breakdown of glycosaminoglycans within lysosomes and rare pathogenic variants within *IDUA* are known to cause accumulation of glycosaminoglycans in lysosomes (mucopolysaccharidosis type I [MPS-1]). People with MPS-1 present with a wide spectrum of complications, such as skeletal deformities or organomegaly, that has been attributed to the variable impact of mutations on enzyme activity, with nonsense mutations causing most severe diseases (Hurler syndrome) (51). Knock-down of *Idua* further led to disturbed bone turn over favoring bone mass build up due to lysosomal overload in osteoblasts (52). While these observations explain bone phenotypes seen for the common cis-pQTL, there are no reports for an elevated risk for inflammatory or autoimmune disease among people with MPS-1 or other evidence from rare variant analysis. Tissue-dependent effects of common variants might be one explanation for the different phenotypes linked to distinct cis-pQTLs for alpha-L-iduronidase. We observed another example of allelic heterogeneity with distinct phenotypic consequences for Alzheimer’s disease and childhood obesity at the 16q22.1 locus (see Supplementary Note 2).

### Phenotypic convergence of rare gene burden and cis-pQTLs

Much effort and funding has been invested into biobank-scale whole-exome sequencing studies (ExWAS) to identify rare deleterious genetic variants and novel disease candidate genes for the development of treatment strategies (53, 54). These studies focused on the rare deleterious end of gene (and protein) dysfunction, but whether common, more subtle, possibly regulatory, effects on the same gene product have similar consequences remains to be established. Such rare to common convergence could, for example, establish dose response relationships to estimate therapeutic windows for drugs (55). We therefore systematically cross-referenced our cis-based phenome-wide colocalization results with a recent exome-wide gene burden study among ˜450,000 UK Biobank participants across almost 4,000 phenotypes (53).

**Figure 6:**
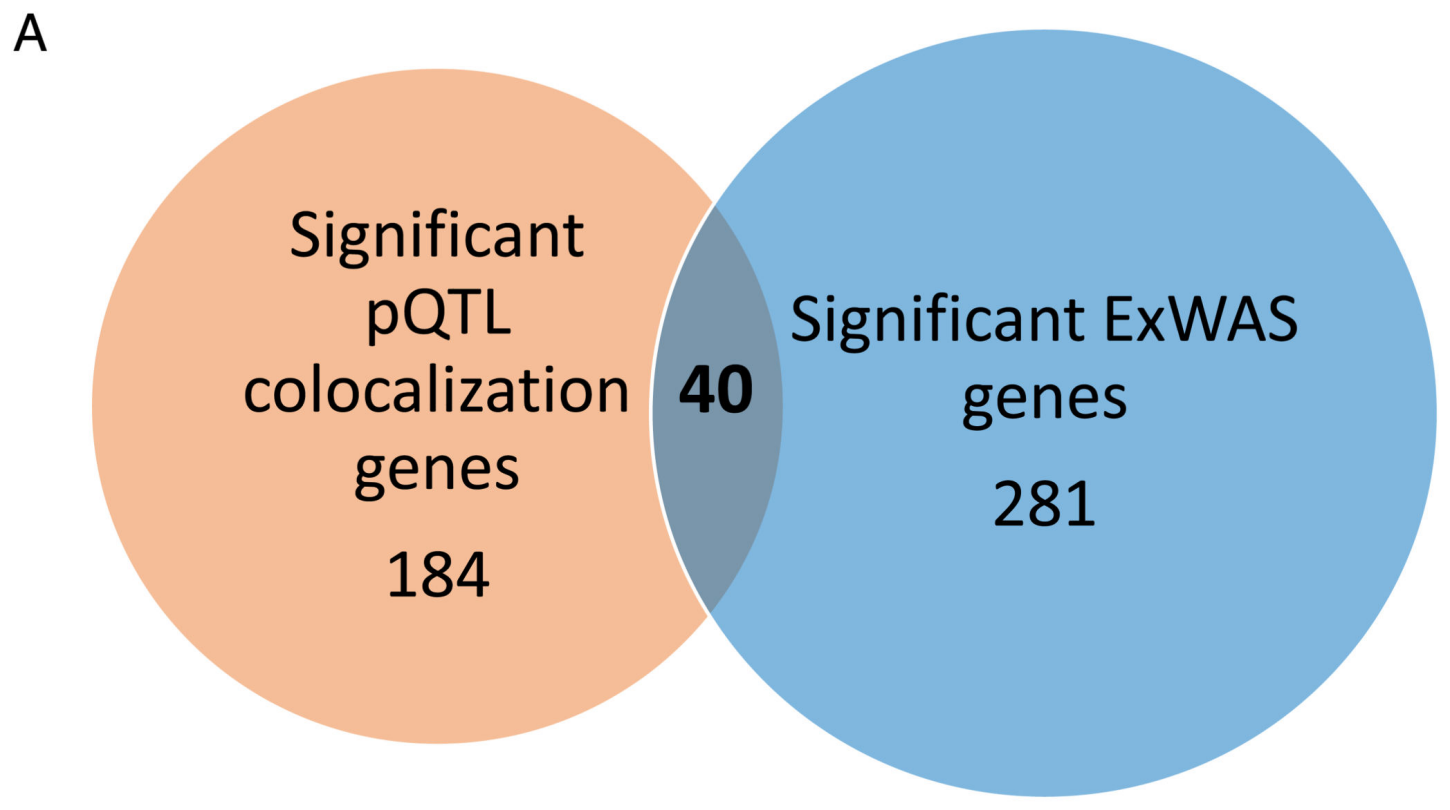

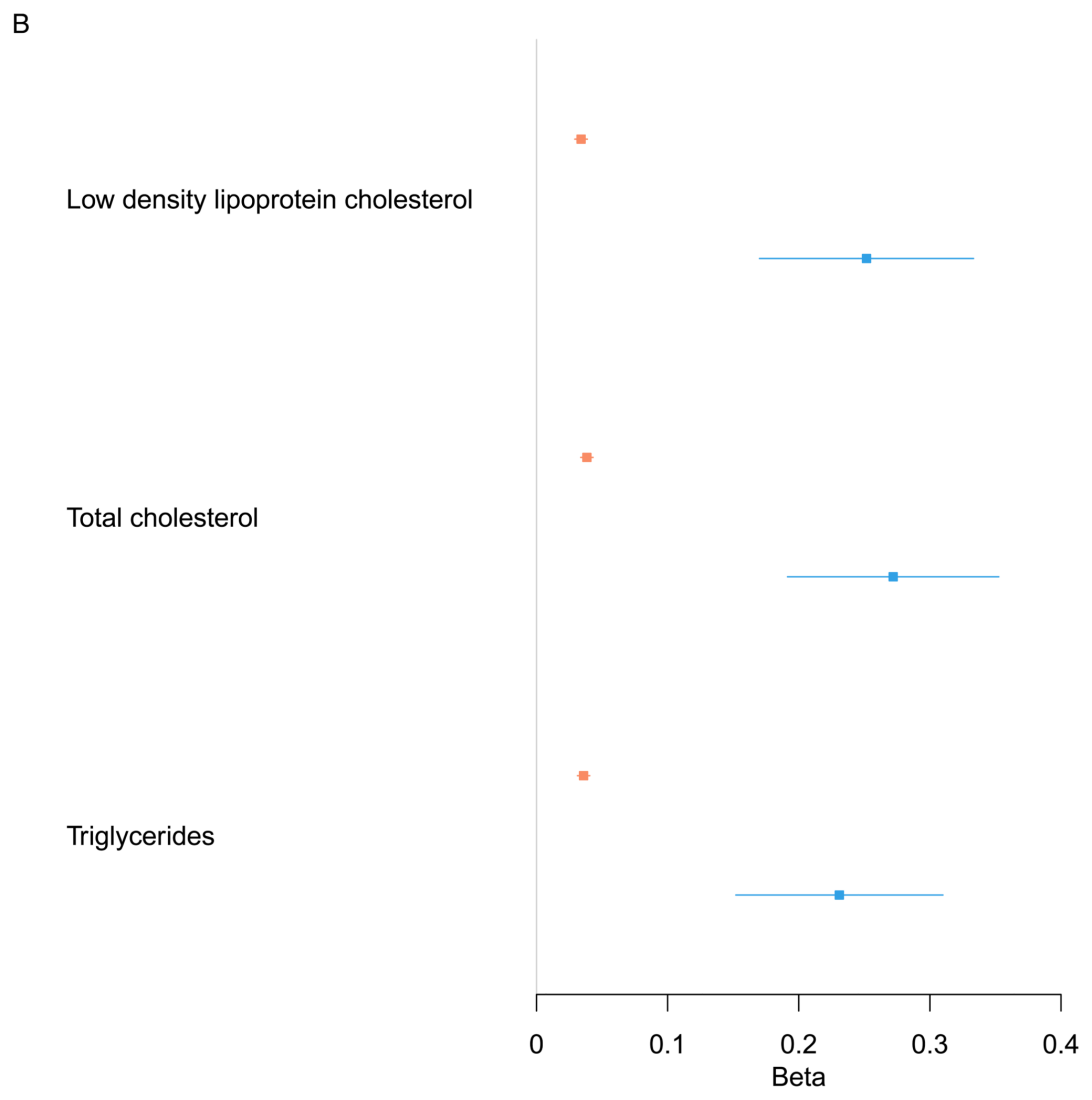

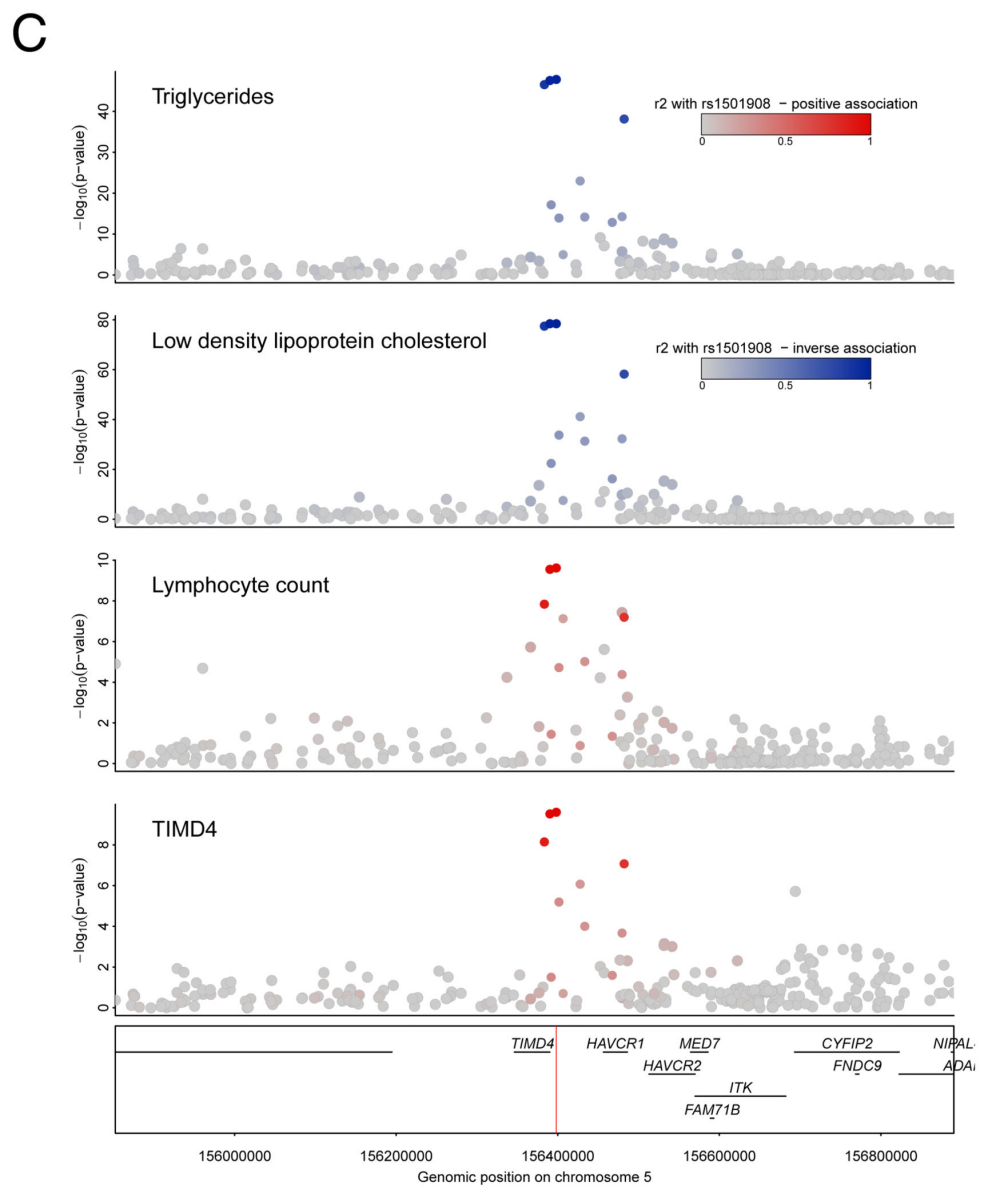
Phenotypic convergence of rare variant burden and common cis-pQTLs for protein coding genes and TIMD4 as an example. A. Venn diagram showing the number of genes with a significant rare variant gene burden association (p<1E-06) with at least one trait (53) in blue and the number of genes with a significant pQTL colocalization (PP>80%) with at least one trait in orange. All 2,939 unique genes covered by Olink Explore 1536 and Explore Expansion assays were investigated. **B. Forest plot comparing the effect size estimates between TIMD4 cis-pQTL (rs58198139) and rare TIMD4 loss of function (LoF) gene-burden results (variant group: missense and loss of function variants with a minor allele frequency < 1%) for low density lipoprotein cholesterol, total cholesterol and triglyceride levels.** Rare TIMD4 loss of function (LoF) gene-burden (n=454,787) results are shown in blue and TIMD4 cis-pQTL associations (n=1,180) are shown in orange. C. **Stacked regional plot of the multi-trait colocalization of TIMD4 cis-pQTL with lymphocyte count, low density lipoprotein cholesterol, and triglycerides.** Red colouring represents a positive effect direction with protein increasing allele with TIMD4 whereas blue represent an inverse association. The hue of the colour represents the strength of r^2^ representing the LD structure, as indicated on the legend. Linear regression models were used to obtain summary statistics presented in this figure.

Among 2,939 protein coding genes analyzed in the present study, 40 (1.3%) showed evidence for phenotypic associations with a rare variant gene-burden (p<1x10^-6^) and statistical colocalization (PP>80%) with a cis-pQTL, whereas 281 and 184 protein coding genes were linked to phenotypes through ExWAS or cis-pQTLs only, respectively (Fig. 6A). Out of the 40 overlapping genes, we observed phenotypic convergence for only 12 genes across 21 phenotypes following manual review to harmonize phenotype definitions (Supplementary Tab. 9). These results clearly exemplify the complementary nature of both approaches and the unique ability of bespoke proteogenomic experiments to prioritize disease mediators and hence putative therapeutic targets.

We observed a dose-response relationship between putative functional consequences for T-cell immunoglobulin and mucin domain containing 4 (TIMD4) and LDL-cholesterol as well as total triglyceride, but not HDL-cholesterol levels in blood (Fig. 6C). The protein-decreasing T-allele of the lead cis-pQTL (rs58198139) was associated with moderate effects on LDL-cholesterol in UK Biobank (MAF=0.26; beta_LDL_=0.03, p-value_LDL_=7x10^-44^), likely mediated by altered protein expression, while the cumulative burden of rare loss-of-function variants was associated with substantially higher LDL-cholesterol levels (beta_LDL_= 0.25, p-value_LDL_= 1.51x10^-9^, variant mask: predicted loss of function and deleterious missense variants with MAF<1%; Fig. 6B). This finding is in line with the locus being one of the earliest discovered loci for polygenic dyslipidemia but with few functional insights gained since (56). TIMD4 is best known for its role in tissue-dependent macrophage efferocytosis of apoptotic cells (57, 58) but does also participate in T-cell activation and recruitment (59). Accordingly, *Timd4^-/-^* mice show impaired macrophage phagocytosis and increased lymphocyte cell counts, an observation recapitulated by our phenome-wide colocalization analyses identifying an inverse association for the protein-decreasing T-allele for lymphocyte counts (beta=1.02, p-value=1.4x10^-12^) with high certainty (PP = 97.5%). Circulating leucocytes and resident M2 macrophages can take up cholesterol from circulating LDL particles and sequestered lipoproteins in the vasculature, but classical pathways, like the LDL-receptor mediated uptake, were shown, at least in mice, to have no substantial effect on plasma LDL-cholesterol levels (60). In contrast, more recent work demonstrated the ability of TIMD4+ adipose tissue macrophages to significantly contribute to the regulation of post-prandial HDL-cholesterol levels in mice (61). While there was no difference in triglycerides or non-HDL cholesterol following TIMD4 blockade, TIMD4 blockade inhibited LDL-induced lysosomal activity *in vitro*, suggesting a role for TIMD4 in peripheral LDL cholesterol processing. These findings provide evidence of a role for TIMD4 in the regulation of systemic lipoprotein metabolism, and taken together with our proteogenomic findings, provide a compelling rationale to explore the role of TIMD4^+^ macrophages in systemic LDL cholesterol metabolism. We note that we did not observe strong genetic evidence for an association between rare or common genetic variation at *TIMD4* for coronary artery disease (cis-pQTL: protein decreasing T-allele, OR [95% CI] = 1.04 [1.02-1.07], p-value=0.002; gene burden: OR [95% CI] = 1.3 [0.90-1.88], p-value=0.16), which may weaken the expectation that pharmacological modulation of TIMD4 could address the residual burden of CAD despite lipid-lowering treatment.

## Discussion

Proteogenomic approaches have the potential to establish a direct link from rare and common variation in or close by protein-encoding genes to human health via the protein product (5–18). Despite recent advances and early successes, the field is still in its infancy with respect to the scale and protein capture, with existing broad-capture technologies currently targeting less than a third of all proteins encoded in the human genome (5–18), not capturing posttranslational modifications and not providing absolute protein quantification.

Here, we identified more than 200 unreported cis-pQTLs by capitalizing on recent assay developments. The fact that we identified hundreds of cis-pQTLs for proteins that have been investigated in studies much larger than ours might be best explained by the need to develop further orthogonal methods to measure protein targets as we have outlined previously (14).

We demonstrate that systematic application of cis-pQTLs to large-scale genetic studies of human diseases can 1) guide causal gene annotation at GWAS loci (e.g., *DKKL1* for multiple sclerosis), 2) identify pathways that link genes to diseases guided by a protein-phenotype network, and 3) complement gene-burden testing of rare variants to discover novel biology. We highlight specific examples in more detail and share a large number of high-confidence protein–phenotype associations that provide a direct guide for functional follow-up and future investigations of variant protein with disease relevance about which little is known to date.

The vast majority (˜90%) of genetic variants identified in GWASs reside in non-coding regions of the genome (62), creating a challenge for variant-to-function annotation. While large-scale and tissue-resolved gene expression studies have been pivotal for gene assignment (2, 63, 64), we, in line with previous studies, demonstrated the efficiency and ability of cis-pQTLs to prioritize causal candidate genes including reassignments at 40% of overlapping loci. In contrast to other annotation approaches, the value of the integration of proteogenomic studies lies in the instrumentalization of the likely biological effector molecules. Studies using proteomic profiling in disease relevant tissues or single cell-types are needed to further elucidate the mechanisms underlying the many thousands of unassigned GWAS loci, including intracellular signaling pathways that cannot be readily proxied from blood samples. The same applies to the incomplete coverage of the circulating proteome, that currently prohibits the distinction of pleiotropic regulatory variants from genuine specific cis-pQTLs.

This study is a powerful demonstration that even moderately sized proteomic studies can result in the identification of novel biology when combined with bespoke analysis pipelines designed for the identification of cis-pQTLs and systematic integration and follow-up with disease GWAS summary statistics. Eventually multiple different technologies will be needed at scale to capture not only proteins of interest but also the vast spectrum of proteoforms with possible distinct phenotypic consequences (14). This prediction is supported by power calculations of the UKB-PPP (17), but also based on the observation that our study identified cis-pQTLs for genes that are under less evolutionary constraint as indicated by higher observed/expected scores for missense (+0.15; p-value=5.0x10^-50^) and loss-of-function (+0.22; p-value=7.5x10^-51^) variation in gnomAD (65). An observation is in line with recent findings among eQTL studies (66).

We observed convergence of gene–phenotype associations between ExWAS and our proteogenomic approach only at a small number of genes. Gene identification with overlapping or converging evidence, as shown for *TIMD4*, provides high confidence about the underlying causal gene, while the incomplete overlap clearly indicates the complementary nature of both approaches for drug target prioritization. An important distinction between both approaches, beyond the different genetic variants covered, is the ability of proteogenomics to emulate protein variation across the whole spectrum of abundance and in some cases function, and not only putative loss-of-function (rarely gain of function), which might explain differences seen in phenotypic consequences between both approaches. In addition, in terms of practicality, integration of pQTLs into colocalization and GWAS loci annotation enabled us to uncover unreported disease biology with a small sample size of 1,180 individuals, whereas substantially larger sample sizes, even millions of individuals, are needed to reach enough power to detect rare variant associations in ExWAS studies for disease endpoints (67).

Our study has some limitations that need to be considered. Affinity-based reagents allow for the quantification of protein abundance but are inherently limited to quantify the level of activity, although a general correspondence between the two can be assumed. This limits insights about the role of protein targets using a proteogenomic approach. Further, numerous posttranslational modifications can change the function and abundance of proteins but are currently not distinguishable using affinity reagents at scale. We deliberately decided to restrict genetic analysis of protein targets to the corresponding protein coding regions (±500kb) for two reasons: 1) the high biological prior to identify genetic variants directly linked to protein function/abundance, and 2) to increase power for statistical analysis by limiting the multiple testing burden. We, however, made genome-wide association statistics based on inverse variance weighted meta-analyses across both sets available to enable targeted discoveries for selected proteins. Larger studies to explore the spectrum of trans-pQTLs and longitudinal studies to explore temporal effects of pQTLs are also needed to better understand the impact of proteins on human health.

In summary, we demonstrate the clear potential of broad-capture proteogenomic studies to identify novel biological pathways that link protein-encoding genes to human (metabolic) diseases. Systematic integration of human genomic with proteomic and phenomic data enables such investigation even in relatively moderately sized studies and can help to prioritize targets and indications for the development of safe and effective therapeutic interventions.

## Materials and Methods

### Study participants

We measured protein levels among 1,200 participants of the European Prospective Investigation into Cancer (EPIC)-Norfolk study, a cohort middle-aged, individuals from the general population of Norfolk, a county in Eastern England which is a component of EPIC (21). We excluded individuals who were related, of non-European descent or did not have a high-quality proteomic profile resulting in 1,180 and 1,178 for genetic analyses for Olink Explore 1536 and Explore Expansion platforms, respectively (Supplementary Tab. 1). The study was approved by the Norfolk Research Ethics Committee (ref. 05/Q0101/191) and all participants gave their informed written consent before entering the study. Information on lifestyle factors and medical history was obtained from questionnaires as reported previously (21). For cost-efficient proteomic profiling, our study consisted of a random sub-cohort (n=755) and a case cohort (n=425) covering a total of seven incident diseases (Supplementary Tab. 2). We performed a replication study in a total of 1707 participants, again divided into a random subcohort (n=1001) and case cohort (n=706) applying the same exclusion criteria as mentioned above (Supplementary Tab. 1–2).

### Proteomic profiling

We used serum samples from the baseline assessment (1993 - 1997) that had been stored in liquid nitrogen for proteomic profiling using the Olink Explore 1536 and Explore Expansion platforms targeting 2925 unique proteins by 2943 assays, of which 2923 unique proteins mapped to a protein encoding locus in genome assembly GRCh37. Details regarding the assay have been described in detail (68). Briefly, proteins are targeted by two separate unique antibodies, each of which are labelled with complementary single stranded oligonucleotides (proximity extension assays (69)). These proximity extension assays hybridization occurs subsequent to the binding of antibody pairs with complementary oligonucleotides which can be quantified using next generation sequencing (NGS). NGS read-outs undergo quality control procedures where internal (incubation, extension and amplification controls) and external (negative, plate and sample controls) controls are included. Normalized protein expression (NPX) units are generated by normalization to the extension control and further normalization to the plate control and reported on a log2 scale. We excluded samples which were extreme outliers using principal component analysis from their entire proteomic profiles. For downstream genetic analysis (fine-mapping and region-based association analysis), we first rank-inverse normal transformed NPX-values to achieve robust statistical analyses with comparable effect estimates across proteins. We then corrected inverse rank transformed values for age, sex, measurement plate, and the first ten genetic principal components using linear regression models. The residuals of this analysis were used throughout the study. For simplicity, we use the term ‘protein levels’ to refer to the relative assay readouts for each protein, although we acknowledge that affinity-based reagents might be affected by genetic or posttranslational modifications of epitope regions.

### Genotyping

EPIC-Norfolk samples (n=21,448) were genotyped on the Affymetrix UK Biobank Axiom array chip by Cambridge Genomic Services, University of Cambridge, UK. Sample and variant QC followed the Affymetrix Best Practices guidelines. Samples were excluded based on DishQC < 0.82 (fluorescence signal contrast), call-rate <97%, heterozygosity outliers and sex discordance checks. Variants were excluded if call-rate <95% or HWE<=1e-6. Monomorphic variants and those with cluster problems detected using Affymetrix SNPolisher were excluded. Genotype imputation was performed using two different reference panels, the Haplotype Reference Consortium (HRC) (release 1) reference panel and the combined UK10K+1000 Genomes Phase 3 reference panel. After pre-imputation QC, 21,044 samples remained for imputation. All SNPs imputed using the HRC reference panel were included, and additional variants imputed using only the UK10K+1000 Genomes reference panel were added to create a combined imputed set. Variants with imputation quality INFO < 0.4 or MAF of < 0.0001 were excluded. All positions are on genome assembly GRCh37. After excluding ancestry outliers, individuals without a high-quality proteomic profile for each panel and pruning the sample set for related individuals, 1,180 and 1,178 individuals were included in proteogenomic analyses for Olink Explore 1536 and Explore Expansion platforms, respectively.

### Fine mapping

We used statistical fine-mapping as implemented in the ‘sum of single effects’ model (SuSiE) using individual level genotype and protein data to identify credible sets at protein encoding loci (±500kb). Briefly, SuSiE employs a Bayesian framework for variable selection in a multiple regression problem with the aim to identify sets of independent variants each of which likely contain the true causally underlying genetic variant (70). We implemented the workflow using the R package *susieR* (v.0.11.92) and default prior and parameter settings. However, we noticed that SuSiE sometimes reports overlapping credible sets or credible sets that contained variants in high LD with already selected ones. Therefore, we adopted a grid search by first iterating the maximum number of credible sets from 2 to 10 (*L* in SuSiE terminology) and subsequently selecting the output for the maximum *L* so that none of the credible sets reported variants in LD (r^2^>0.1). We further tested for independent effects of all lead credible set variants (selecting using highest posterior inclusion probability) by including them in a joint regression model. We only took forward credible sets which were identified through fine-mapping and also met stringent genome-wide (p<5x10^-8^) significance in linear regression models that jointly modelled the effect of lead credible variants in the locus. In sensitivity analysis, we did not saw an effect of the time passed since storage on the identified cis-pQTLs. We used R v.3.6.0 to compute regression models.

### Testing for effect modification by sex

In order to test for potential differences of the cis-pQTLs identified in this study, we included an interaction term between the cis-pQTL and sex in a linear regression model with the same adjustments as in the main analysis. We have defined significance at a Bonfferoni-corrected threshold (p<0.05/1553).

### Replication of cis-pQTLs

We tested for independent replication of identified lead variants from credible sets by running the exact same joint models in a separate set of EPIC-Norfolk (n=1707) for which proteome profiling was done at a later timepoint. We considered cis-pQTLs to replicate, if they showed directionally concordant associations and further met a stringent Bonferroni-corrected threshold of significance (p<3.21x10^-5^). A total of 1,506 out of 1,553 (96.9%) cis-pQTLs fulfilled these criteria. We further observed a strong correlation (r=0.96) between the effect size estimates between the two studies (Supplementary Table 3).

### Region-based association testing

To complement fine-mapping analysis, we computed regional association statistics at protein coding loci (±500kb) using fastGWA software provided by GCTA (v. 1.93.2beta) (71). We used residuals from rank-inverse normal transformed NPX-values corrected for age, sex, plate effect and the first ten genetic principal components. To account for the different selection designs of the sub-cohort and the cases, we performed these analyses within each cohort separately and combined in an inverse-variance fixed-effects meta-analysis in METAL (72).

### Gene, Variant, and Protein annotation

We obtained conservation scores for all protein coding genes from gnomAD. We used the Variant Effect Predictor software (73) (version 98.3) with the --pick option to annotate all independent lead variants and proxies (r^2^>0.6) of identified pQTLs in our data set and report possible functional consequences. We collapsed pQTLs mapping to the same functional variant to reduce redundancy. We further obtained protein characteristics, e.g., glycosylation sites, from UniProt (74). To test for enriched characteristics of proteins among those with at least one cis-pQTL, we performed Fisher’s exact test using all proteins captured by the Olink Explore and Expand platform as a background.

### Variance explained

We estimated the variance explained by pQTLs for protein levels of protein with at least one cis-pQTL by including all cis-pQTLs in a linear regression model using residual protein levels as outlined in the region-based association testing section. We used the R^2^ of the entire model as an estimate for the variance explained.

### Annotation of GWAS catalog loci

We downloaded genome-wide significant summary statistics from the GWAS catalog (date 23/03/2022; (1)) and tested whether any of the lead credible set variants (lead cis-pQTLs) or proxies (r^2^>0.8) with the lead cis-QTLs have been reported to be associated with any non-proteomic trait, that is omitting any results that related to multiplex proteomic assays. Out of 347,165 entries (n=9,997 unique traits), 212,628 entries (n=5,391 unique traits) passed this and additional filtering steps (missing effect estimates, missing risk allele, and not passing genome-wide significance). For each cis-pQTL – GWAS variant mapping, we compared the reported or mapped gene (closest gene assigned by the GWAS catalog) to the protein-encoding gene at the locus.

### Phenome-wide analyses at protein-encoding loci

We performed phenome-wide analyses using statistical colocalization for 914 protein targets where we had evidence for at least one cis-pQTL. To this end, we queried the Open GWAS database (75, 76) using a defined region (±500 kb) around the protein-encoding gene body and tested whether any of the traits in the databases showed a high PP of shared genetic signal with plasma concentrations of the encoded protein target using statistical colocalization (77). We only tested phenotypes for colocalization that had at least suggestive evidence of association (p<10^-6^) with the lead cis-pQTL in the region or a close proxy (r^2^>0.8). We chose a cut-off of PP>80% to declare that a protein target and a phenotypic trait are highly likely to share a genetic signal at a locus. As there are currently no methods to control the false discovery rate for colocalization screens, we used a conservative prior setting with p_12_=1x10^-6^ and further ensured that regional sentinel variants were in strong LD (r^2^>0.8). To avoid spurious colocalization results due to imperfect overlap of SNPs, we filter all results for which the strongest cis-pQTL or sufficient proxy (r^2^>0.8) in the overlapping set was not included in the overlapping set of SNPs or if less than 500 SNPs were overlapping. We used the *igraph* package in R to visualize protein – disease colocalization results as a network to account for cross-disease dependencies established by proteins. In studies where linear regression was used for binary traits for computational efficiency, we used the following formula to report ORs where needed: log(odds ratio) = β / (μ * (1 - μ)), where μ = case fraction.

### Incorporation of gene expression data

We systematically tested for a shared genetic signal between plasma abundances of a protein and gene expression levels (eQTL) of the protein coding gene in 49 tissues from the GTEx project (v8) (78). We used a similar colocalization framework as described above but adopting a less stringent P_12_ prior (p_12_=1x10^-5^) to account for the higher biological prior of genetic signals in the protein encoding region. All GTEx variant-gene cis-eQTL associations from each tissue were downloaded in January 2020 from https://console.cloud.google.com/storage/browser/gtex-resources.

### Phenotypic convergence between pQTL colocalization and rare loss of function gene-burden associations

To compare the phenotypic convergence of rare loss of function gene-burden and cis-pQTLs colocalization results, we downloaded single variant and gene-burden results for 3,986 phenotypic outcomes from UK Biobank respectively which were analysed by Backman et al. (2021) (downloaded on: 07/12/2021); (53). We filtered the results for 2,939 protein coding genes covered by the Olink Explore 1536 and Explore Expansion platforms. We compared the phenotypic convergence of genes that were significant for at least one phenotypic outcome in the exome-wide association analysis at exome-wide significance (p<1x10^-6^) with the pQTLs that showed significant statistical colocalization for at least one trait (PP>80%). If ExWAS results were significant for more than one variant group for the same gene – trait association, we have filtered the results to only take forward the most significant finding.

### Multitrait colocalisation

We used hypothesis prioritisation in multi-trait colocalisation (HyPrColoc) (79) at selected protein loci to identify a shared genetic signal across various traits, including gene expression, plasma protein levels, and prioritized phenotypes from the disease-wise colocalization framework. HyPrColoc provides for each cluster three different types of output: 1) a PP that all phenotypes in the cluster share a common genetic signal, 2) a regional association probability, that it, that all the phenotypes share an association with one or more variants in the region, and 3) the proportion of the PP explained by the candidate variant. We considered a highly likely alignment of a genetic signal across various phenotypes if the PP>80% and report obtained PPs otherwise.

## Supplementary Material

Supplementary Materials

Supplementary Tables

## Data Availability

The EPIC-Norfolk data can be requested by bona fide researchers for specified scientific purposes via the study website (https://www.mrc-epid.cam.ac.uk/research/studies/epic-norfolk/). Data will either be shared through an institutional data sharing agreement or arrangements will be made for analyses to be conducted remotely without the need for data transfer. Fine-mapped summary statistics for protein coding regions can be found here: https://doi.org/10.5281/zenodo.7576293. The genome-wide summary statistics resulting from the meta-analysis between discovery and replication samples (n = 2,887) can be downloaded for all protein targets included in this study from https://omicscience.org/. Genome-wide association studies for anthropometric phenotypes have been conducted using the UK Biobank resource (application no. 44448). Access to the UK Biobank genotype and phenotype data is open to all approved health researchers (http://www.ukbiobank.ac.uk/). GWAS Catalogue summary statistics (v.1.0.2) were downloaded (March 2022) from https://www.ebi.ac.uk/gwas/api/search/downloads/studiesalternative. All GTEx variant-gene cis-eQTL associations from each tissue were downloaded (January 2020) from https://console.cloud.google.com/storage/browser/gtex-resources. OpenGWAS summary statistics were accessed via *ieugwasr* package in R v3.6.0. Associated code and scripts for the analysis are available on GitHub (https://github.com/MRC-Epid/pGWASOlinkEPIC).
