# Supplementary material for: Proteogenomic links to human metabolic diseases
Source: Nat Metab. Author manuscript; Available in PMC 2023 Aug 12. (PMC7614946; doi:10.1038/s42255-023-00753-7)
Supplement: Supplementary Materials [file EMS175897-supplement-Supplementary_Materials.pdf]

Supplementary Materials for

Proteogenomic links to human metabolic diseases

Mine Koprulu[1*], Julia Carrasco-Zanini[1], Eleanor Wheeler[1], Sam Lockhart[1,2], Nicola D. Kerrison[1], Nicholas Wareham[1], Maik Pietzner[1,3], Claudia Langenberg[1,3,4]

Supplementary Notes

Supplementary Note 1: *DKKL1* as a candidate causal gene for Multiple Sclerosis.

Multiple Sclerosis (MS) is an autoimmune, inflammatory, and neurodegenerative disease of the central nervous system that is caused by both genetic and strong environmental factors (1). A strong signal at 19q13.33 is one of 233 reported GWAS loci (2). Several variants in high LD (r^2>0.9) reported for this locus have been linked to different candidate causal genes, including *DKKL1*, *CD37*, and *SLC6A16* which was the most recently annotated gene based on a sophisticated ensemble of methods (2) . We identify a shared genetic signal (PP=96.1%, **Supplemental Fig. 1**) between dickkopf like acrosomal protein 1 (DKKL1), encoded by *DKKL1*, and MS at this locus, led by a cis-pQTL (rs2288480; MAF=0.25) in high LD (r^2=0.97) with the lead MS variant (rs1465697; MAF=0.33, OR=1.09, p-value=$3x10^{-18}$). We note that the lead cis-pQTL was also in LD (r^2=0.97) with a recently identified variant at the same locus for systemic lupus erythematosus among East Asians (3).

The lead cis-pQTL is in strong LD (r^2>0.8) with a cluster of three common missense variants (rs2288481, rs2303759, and rs1054770) that might impair protein function or processing. Little is known about the biological role of DKKL1 in general, but a non-essential role in spermatogenesis has been described (4). However, a link towards MS and/or SLE might be conceivable via a possible role of DKKL1 in adaptive immunity and hence the inflammatory component of MS. Briefly, *DKKL1* expression is enriched among memory B-cells (5) and an independent secondary cis-pQTL (rs66532151, MAF=22.4%) for DKKL1 tagged (r^2>0.96) a cluster of variants associated with different characteristics of $CD20^+$ memory B-cells (6). This cis-pQTL (rs66532151) was associated with MS at p-value=$3.4x10^{-7}$, providing late genetic evidence for depletion of B-cells being one of the most effective treatments for MS, a therapeutic strategy that originally emerged from clinical and neuropathological studies (7, 8). Further follow-up studies are needed to clarify effect strengths, a possible role of DKKL1 in immune cells, whether DKKL1 may play a role in B-cell hyperactivity observed in MS (7).

Supplementary Note 2: Allelic heterogeneity at interleukin 34 (IL-34) loci translates into distinct phenotypic consequences.

We discovered three distinct credible sets for plasma levels of interleukin 34 (IL-34) at 16q22.1. Two contained independent (r^2=0.07) lead cis-pQTL variants with distinct structural consequences on the protein, that were also associated with two distinct outcomes – Alzheimer's disease (9) and childhood obesity (10) (**Supplementary Figure 2**). rs4985556 is associated with increased risk for Alzheimer's disease (MAF=12.2%; beta=0.07, p-value=2.3×10^{-8}) and introduces a premature stop (p.Tyr213Ter), truncating the protein and likely affecting dimerization and possibly secretion. rs8046424 (alternate allele: C; r2=0.96 with lead sentinel childhood obesity variant rs4985555) is associated with reduced childhood obesity (MAF = 48.2%; beta=-0.008, p-value<4.4×10^{-9}). It is a missense variant (p.Glu123Gln) of moderate consequence (CADD score 11.6) that maps to a binding domain of the cognate IL-34 receptor CSF-1R (11). Therefore, both variants will likely strongly (rs4985556) or moderately (rs8046424) attenuate signalling via CSF-1R, which has been shown to drive cerebrovascular pathologies that are common in Alzheimer's disease (12). While the gradient of structural consequences translates into a graded effect on Alzheimer's disease (rs8046424, beta=0.03, p=3.6×10^{-4}), the absence of any effect of the more detrimental variant (rs4985556) on childhood obesity (beta=-0.001, p=0.59) might point to a different, yet to be defined, pathway.

Supplementary Figures

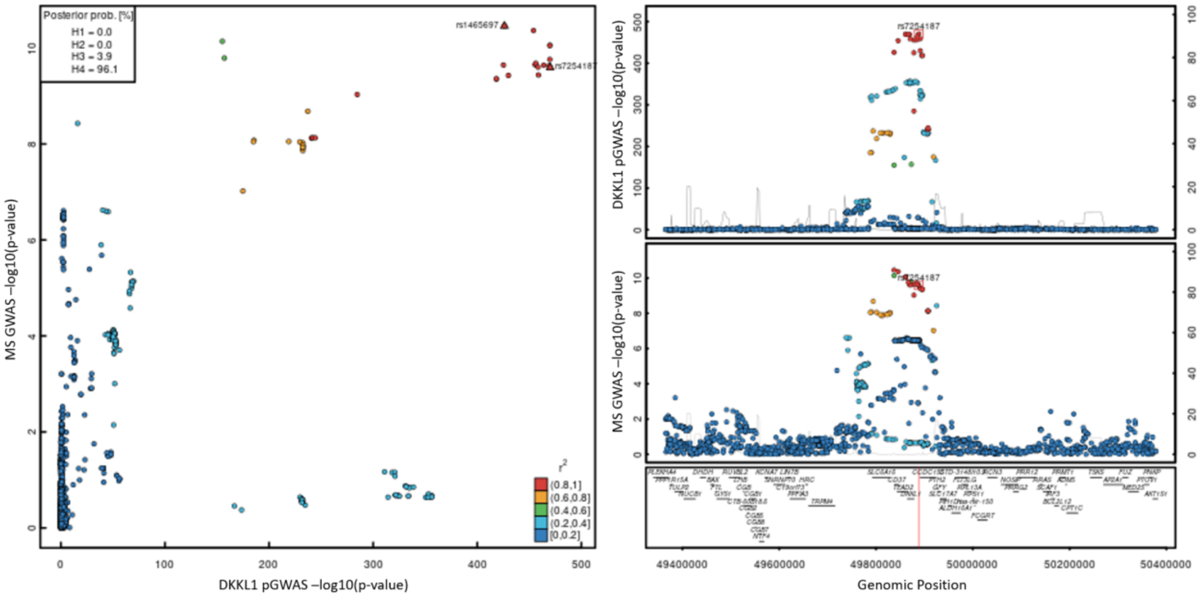

Supplementary Figure 1: Locusplot comparing association statistics for DDKL1 and multiple sclerosis (MS). The left panel displays a comparison of -$\log_{10}$-transformed p-values from GWAS summary statistics for genetic variants in a 500 kb region around the lead signal on chromosome 19. Colouring was done based on linkage disequilibrium with the lead variant for the protein. The right panel is a stacked locuszoom plot (DKKL1 on top, MS on the bottom) with annotation of protein-encoding genes underneath. Location of the lead variant is indicated by a red line. Linear regression models were used to obtain summary statistics presented in this figure.

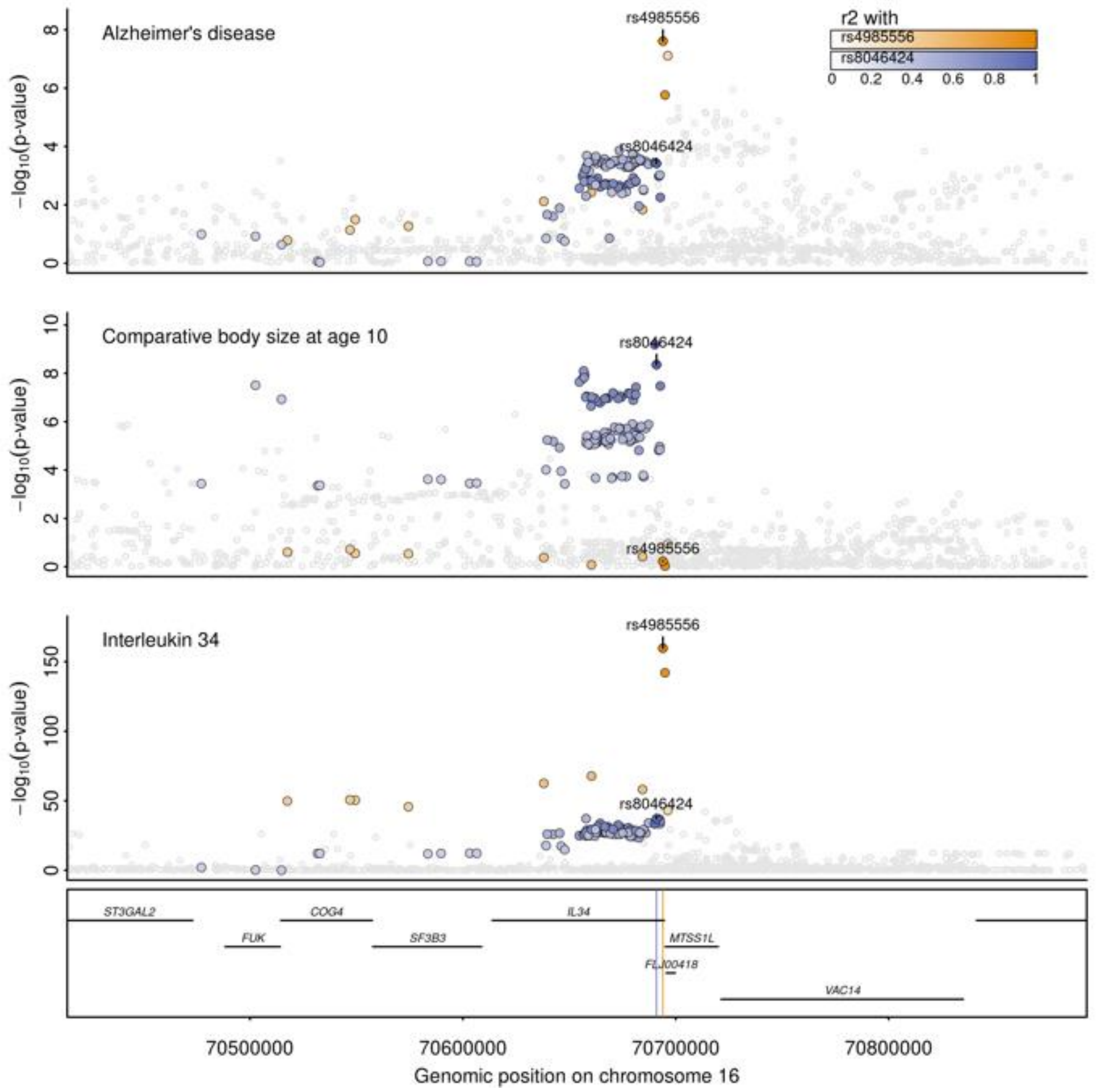

Supplementary Figure 2: Allelic heterogeneity at protein coding loci translates into distinct phenotypic consequences at *IL34*. Regional association plots centered around *IL34* (±200kb) for plasma interleukin 34 levels, comparative body size at age 10 (10), and Alzheimer's disease (9). Shown are association statistics (p-values) from genome-wide association analysis. Single genetic variants were coloured based on LD with two distinct cis-pQTLs (rs4985556 – orange; rs8046424 – purple). Linear regression models were used to obtain summary statistics presented in this figure.

Supplementary Table titles

Supplementary Table 1. Demographics of the European Prospective Investigation into Cancer (EPIC)-Norfolk study.

Supplementary Table 2. List of diseases for discovery (n=425) and replication (n=706) cases. Please note that some individuals had more than one disease.

Supplementary Table 3. Independent credible sets for protein targets at protein encoding loci (±500kb) identified by fine-mapping. This table includes all 1,553 independent credible sets for protein targets, annotated by the lead variant in each credible set and their functional annotations.

Supplementary Table 4. Variance explained by all cis-pQTLs for each protein with at least one cis-pQTL identified in this study.

Supplementary Table 5. Results from colocalization analysis with gene expression trait loci (eQTLs) from the GTEx version 8 release at the protein encoding loci (±500kb). The table contains all gene expression - protein pairings where the posterior probability of a shared signal was above 80%. For each protein target where we have observed strong statistical colocalization (posterior probability of shared signal > 80%), all of the significant tissues are listed, and relevant posterior probabilities are provided in brackets.

Supplementary Table 6. cis-pQTLs with sex differential effects at a Bonferroni corrected significance threshold (p<0.05/1553).

Supplementary Table 7. Protein-target – phenotype pairs with strong evidence of colocalization at the protein encoding locus (±500kb). The table contains all protein – phenotype connections where the posterior probability of shared signal was above 80% and regional sentinel variants were in strong linkage disequilibrium (r^2>0.8). Information on draggability based on common gene entries from Finan et al. (2017) (13).

Supplementary Table 8. pQTL mapping of candidate causal genes at previously reported GWAS loci from GWAS Catalog. For each mapping cis-pQTL – GWAS variant pair all curated traits are listed, and two columns indicate whether the protein-encoding genes has been reported as the causal gene at this locus or is the closest gene at this locus.

Supplementary Table 9. Phenotypic convergence between pQTL colocalization and rare loss of function gene-burden associations. This table includes information on the phenotypic convergence between pQTL colocalization and rare loss of function gene-burden associations from Backman et al (2021) (14) for 40 overlapping genes. Following manual review to harmonize phenotype definitions, converge flag column indicates the phenotypic convergence amongst the list of phenotypes for 40 overlapping genes. Information on draggability based on common gene entries from Finan et al. (2017) (13).

Supplementary Data

Supplementary Data 1: Protein – disease network as Cytoscape session. Results from phenome-wide colocalization at protein coding loci (±500kb) are shown. For simplicity, only proteins with at least one binary outcome (i.e., mainly diseases) association are included. Proteins are presented with a square, binary outcomes are presented with large circles, and continuous outcomes are presented with small circles. The colour for the circles present the trait category. Edges between proteins and phenotypes represent strong evidence for a shared genetic signal (PP>80% and LD between regional sentinel variants >0.8). Effect directions are indicated by the line type (solid = higher protein abundance, increased risk, dashed = higher protein abundance, reduced risk) and derived based on the lead cis-pQTL at the corresponding locus. The full list of colocalization results can be found in Supplementary Table 7. Abbreviations: GIT, gastrointestinal tract.

References

1. Filippi M, Bar-Or A, Piehl F, Preziosa P, Solari A, Vukusic S, et al. Multiple sclerosis. Nat Rev Dis Primers. 2018;4(1):43.
2. Consortium IMSG. Multiple sclerosis genomic map implicates peripheral immune cells and microglia in susceptibility. Science. 2019;365(6460).
3. Yin X, Kim K, Suetsugu H, Bang SY, Wen L, Koido M, et al. Meta-analysis of 208370 East Asians identifies 113 susceptibility loci for systemic lupus erythematosus. Ann Rheum Dis. 2021;80(5):632-40.
4. Kaneko KJ, Kohn MJ, Liu C, DePamphilis ML. The acrosomal protein Dickkopf-like 1 (DKKL1) is not essential for fertility. Fertil Steril. 2010;93(5):1526-32.
5. Uhlen M, Karlsson MJ, Zhong W, Tebani A, Pou C, Mikes J, et al. A genome-wide transcriptomic analysis of protein-coding genes in human blood cells. Science. 2019;366(6472).
6. Orrù V, Steri M, Sidore C, Marongiu M, Serra V, Olla S, et al. Complex genetic signatures in immune cells underlie autoimmunity and inform therapy. Nat Genet. 2020;52(10):1036-45.
7. Cencioni MT, Mattoscio M, Magliozzi R, Bar-Or A, Muraro PA. B cells in multiple sclerosis - from targeted depletion to immune reconstitution therapies. Nat Rev Neurol. 2021;17(7):399-414.
8. Granqvist M, Boremalm M, Poorghobad A, Svenningsson A, Salzer J, Frisell T, et al. Comparative Effectiveness of Rituximab and Other Initial Treatment Choices for Multiple Sclerosis. JAMA Neurol. 2018;75(3):320-7.
9. de Rojas I, Moreno-Grau S, Tesi N, Grenier-Boley B, Andrade V, Jansen IE, et al. Common variants in Alzheimer's disease and risk stratification by polygenic risk scores. Nat Commun. 2021;12(1):3417.
10. Richardson TG, Sanderson E, Elsworth B, Tilling K, Davey Smith G. Use of genetic variation to separate the effects of early and later life adiposity on disease risk: mendelian randomisation study. BMJ. 2020;369:m1203.
11. Liu H, Leo C, Chen X, Wong BR, Williams LT, Lin H, et al. The mechanism of shared but distinct CSF-1R signaling by the non-homologous cytokines IL-34 and CSF-1. Biochim Biophys Acta. 2012;1824(7):938-45.
12. Delaney C, Farrell M, Doherty CP, Brennan K, O'Keeffe E, Greene C, et al. Attenuated CSF-1R signalling drives cerebrovascular pathology. EMBO Mol Med. 2021;13(2):e12889.
13. Finan C, Gaulton A, Kruger FA, Lumbers RT, Shah T, Engmann J, et al. The druggable genome and support for target identification and validation in drug development. Sci Transl Med. 2017;9(383).
14. Backman JD, Li AH, Marcketta A, Sun D, Mbatchou J, Kessler MD, et al. Exome sequencing and analysis of 454,787 UK Biobank participants. Nature. 2021;599(7886):628-34.
